# “Development in well-being and social function among Danish hemophilia patients with HIV: a three-wave panel study spanning 24 years”

**DOI:** 10.1186/s12889-019-8062-9

**Published:** 2019-12-19

**Authors:** Emilie B. Ingvorsen, Christina Schnohr, Terkel Andersen, Lars Lehrmann, Eva Funding, Lone H. Poulsen, Karen B. Holm, Alex L. Laursen, Jan Gerstoft, Jakob B. Bjorner

**Affiliations:** 1 Department of Public Health Section of Social Medicine, University of Copenhagen, Oester Farimagsgade 5, 1014 K Copenhagen, Denmark; 2 The Danish Haemophilia Society, Copenhagen, Denmark; 3 Department of Hematology Rigshospitalet, Copenhagen, Denmark; 4 Aarhus University Hospital, Centre for thrombosis and hemostasis, Aarhus, Denmark; 5 Department of Infectious Diseases Aarhus, Aarhus University Hospital, Aarhus, Denmark; 6 Department of Infectious Diseases, Rigshospitalet, Copenhagen Ø, Denmark; 7 Optum Patient Insights, Johnston, RI USA

**Keywords:** HIV, Hemophilia, Well-being, Stigma, Social conditions, Employment

## Abstract

**Background:**

Between 1975 and 1985 a total of 91 Danish patients with moderate and severe hemophilia (PWH) was infected with HIV constituting a major scandal in the Danish health care system. This study describes the burden of HIV infection among Danish PWH by evaluating changes from 1988 to 2012 in well-being, social function, experiencing stigma and openness about disease among Danish HIV^+^ PWH.

**Methods:**

Three anonymous surveys were conducted in 1988, 2001 and 2012 targeting all Danish patients with moderate to severe hemophilia. Survey responses were received from 53, 21 and 18 HIV^+^ PWH respectively. A matched comparison sample of HIV^−^ PWH was identified for each survey-year, using propensity score matching. Differences for each survey-year and trends over time were analyzed using ordinal logistic regression.

**Results:**

In 1988, HIV^+^ PWH had more psychosomatic symptoms than HIV^−^ PWH, but in 2001 life satisfaction was higher among HIV^+^ PWH than among HIV^−^ PWH. Tests of differences in trend over time showed larger improvements in life satisfaction among HIV^+^ PWH than HIV^−^ PWH, while HIV^−^ PWH showed an increase in educational level compared to HIV^+^ PWH. Analysis restricted to HIV^+^ PWH showed an increase in perceived stigmatization.

**Conclusions:**

Differences between Danish HIV^+^ and HIV^−^ PWH regarding well-being and psychosomatic symptoms seem to have evened out between 1988 and 2012. However, results suggest that HIV^+^ PWH still experience stigmatization and lower levels of education.

## Background

The bleeding disorders Hemophilia A and B, and von Willebrand’s Disease are chronic diseases that requires lifelong and costly treatment. These rare and innate diseases are caused by a deficiency of coagulation factor causing longer bleeds when injured than persons with normal coagulation factors. Classification of hemophilia is based on plasma procoagulant levels, and classified as mild, moderate or severe [1]. In spite of being a rare disease, hemophilia has gained a high level of awareness due to the hemophilia scandal. Before heat-treatment of factor products was introduced in 1984, patients with moderate or severe hemophilia (PWH) were infected with both HIV and Hepatitis C worldwide through contaminated blood products used as part of their hemophilia-treatment. In Denmark, a total of 91 PWH were infected with HIV between 1975 and 1985, in some incidents even after the risks of this treatment became known. Historically, patients with hemophilia (PWH) have been burdened with increased risk of bleeds, increased mortality risk, chronic pain and reduced mobility due to hemophilic arthropathy [2]. Improvements of hemophilia treatment – particularly treatment with factor products - during the1970s were accompanied by expectations that PWH would experience reduced mortality, reduced morbidity and increased well-being. However, the HIV epidemic caused a setback for PWH, as HIV infection had devastating impact on the quality of life, quality of care and longevity of PWH [3]. HIV^+^ PWH were described as one of the major risk groups for AIDS along with intravenous drug users and homosexuals [4]. In the 1980s being infected with HIV was associated with social stigma and both physical and psychological morbidity [5]. At this point in time, there was no effective treatment for HIV and HIV^+^ PWH had a short life expectancy. Many HIV^+^ PWH retired early and more than 20 countries, including Denmark, established compensation programs for the patients infected through contaminated blood products [6, 7]. From the 1980s and to the 2010s the lives of HIV^+^ PWH have undergone improvements in terms of prognosis, disease consequences, and treatment regimens, but also concerning the social stigma around HIV^+^ patients [8, 9]. However, the consequences of these major improvements for well-being among HIV^+^ PWH are not well described [10].

Previous studies have shown varying results when comparing HIV^+^ PWH and HIV^−^ PWH on quality of life (QoL), well-being, social function, and psychosomatic symptoms. Some studies have found increased psychological distress [11–14], although less so for adolescents being open about their HIV status [15]. Other studies found no significant differences between HIV^+^ PWH and HIV^−^ PWH [16–18]. Studies using prospective data regarding the development in well-being and social function are scarce and to the best of our knowledge there have been no recent studies on symptom burden among HIV^+^ PWH.

To evaluate the future needs of PWH and in particular HIV^+^ PWH, the Danish Hemophilia Society collected survey data on all Danish patients with moderate to severe hemophilia in 1988. These surveys were repeated twice, in 2001 and 2012. Spanning 24 years, the data enable us to study the development in well-being, social function, and stigmatization among HIV^+^ PWH.

The present study has three aims: 1) To compare well-being and social function between Danish HIV^+^ and HIV^−^ PWH at three time points, 2) To assess the trend in well-being and social function over time in HIV^+^ as compared to HIV^−^ PWH, 3) to assess the trend over time in stigmatization and openness about HIV for HIV^+^ PWH (these questions were only asked HIV^+^ PWH). We hypothesized a positive development in well-being and social function, as well as reduced stigmatization due to improved treatment and changes regarding the public attitude and knowledge about HIV.

## Methods

### Three-wave panel study

The Danish Hemophilia Society collected survey data through anonymous questionnaires in 1988, 2001 and 2012. The surveys were developed by the Danish Hemophilia Society. The two Danish hemophilia centers located at Aarhus University Hospital and Copenhagen University Hospital Rigshospitalet, identified all Danish patients with moderate-to-severe hemophilia A or B (factor VIII or IX ≤5%) or type 3 von Willebrand’s disease. The centers also registered HIV status. For 1988, there were 85 HIV^+^ PWH out of a total of 212 PWH (6 HIV^+^ PWH were diseased before start of the study). In 2001, there were 30 surviving HIV^+^ PWH out of 190 PWH. In 2012, there were 27 HIV^+^ PWH out of 240 PWH. Since no new cases of HIV infection occurred after 1985, the HIV^+^ PWH registered in 1988, 2001 and 2012 are subsamples of the 91 PWH originally infected. The hemophilia centers distributed the 1988 and the 2001 surveys by mail to all identified PWH. In 2012, the survey was administered online with the opportunity to receive a paper version of the questionnaire. In 1988, 53 responses were received from HIV^+^ PWH out of a total of 135 responses (response rates 62 and 64% respectively). In 2001, 21 responses were received from HIV^+^ PWH out of a total of 164 responses (response rates 70 and 86%). In 2012, 18 responses were received from HIV^+^ PWH out of a total of 166 responses (response rates 67 and 69%).

### Variables

The study variables are presented in Table 1. Data was self-reported except for yearly factor use; severity of hemophilia; inhibitor; hepatitis B and C; and HIV infection. These variables were assessed by self-report in 1988 and 2012 but from patient charts in 2001. Hemophilia severity was not assessed in 1988. Some of the variables are summarized as two scales regarding *joint mobility* and *psychosomatic symptoms*. *Joint mobility* was assessed by five questions on the range of motion in periods with no bleeds and summed into a scale ranging from 0 (reduced mobility for all types of joint) to 5 (full mobility for all types of joint) [19]. *Psychosomatic symptoms* were measured by four questions concerning discomforts within the last 2 weeks: headache, anxiety, depression, and fatigue. The responses to the questions were summed into a scale ranging from 0 (no symptoms) to 4 (symptoms within every category). A detailed description of the questionnaires is provided as Additional file 1.

**Table 1:** Description of study variables

	Variable	Definition/Question
Background and clinical variables	Age	Age at January 1st of 1988, 2001 and 2012
	Number of bleeding episodes treated with factor	1988: 5 response categories 2001–2012: # of episodes
	Yearly factor use^a^	Units per year
	Severity of hemophilia^b^	Moderate; Severe
	Inhibitor (ever)^a^	Never; Current or previous
	Hepatitis B or C (ever)^a^	Never; Current or previous
	HIV Infection^a^	Yes; No
	Joint mobility	Questions on range of motion in periods with no bleeds in the following joints: hips, knees, ancles, shoulders, and elbows
Social function	Education	Highest education completed
	Work	Questions on current employment, work hours and social benefits.
	Family type	Living with spouse or partner; Living alone; Other family type (e.g. living with parents or house sharing)
	Social activities	“Do you attend meeting, clubs, or other activities outside work or school, including sports, evening school or the like?”
Well-being	Life satisfaction	“All in all, how satisfied or dissatisfied are you with your life as it stands today?”
	Psychosomatic symptoms	^c^Headache; ^c^Anxiety, nervousness, unrest; ^c^Depressed, in low spirit, unhappy; ^c^Tiredness.
	Worries	“Patients with hemophilia have a certain risk of developing life-threatening bleeds. Do you ever think about that?”
	Being alone	“Are you ever alone, but want to be together with other people?”
Stigma	Stigma	“How often have you felt like people look down upon you, avoid you or in any way react negatively about your HIV positive status?”
	Openness	“Who knows that you are HIV positive?”

^a^1988 and 2012: Self-reported, 2001: Extracted from charts

^b^1988: not recorded, 2001: Extracted from charts, 2012: Self-reported

^c^ “Have you, within the last 2 weeks, been bothered with following pain or discomforts.”

### Statistical analyses

To enable comparisons between HIV^+^ PWH and HIV^−^ PWH, a matched comparison sample was identified for each of the 3 years, using propensity score matching [20]. Matching variables were age group, yearly factor use and hepatitis infection. Hepatitis infection was not used as matching variable in 1988, since a large proportion of patients in 1988 did not know whether they had been infected. After controlling for age, factor use and hepatitis, other background variables (number of bleeding episodes, presence of inhibitor and joint mobility) were not significantly associated with HIV status and were not used as matching variables. The probability of being HIV infected was calculated from age, factor use, and hepatitis using a logistic regression model. HIV^+^ PWHs were matched with HIV^−^ PWH of similar risk for HIV infection using 1:1 optimal matching (R package MatchIt [21]). Comparisons between HIV^+^ and HIV^−^ PWH were conducted for each year using ordinal logistic regression. Comparison of HIV^+^ PWH and HIV^−^ PWH showed that the propensity score matching did not provide a perfect match on age and factor use in 1988 and 2012. These variables were therefore included as covariates in subsequent statistical testing. Trend over time was evaluated in a combined data set by including a year times HIV interaction in an ordinal logistic regression model. Comparisons between 1988, 2001 and 2012 were done at group level, since the anonymous questionnaires did not permit tracing of individual patients.

## Results

### Treatment related variables and joint mobility

The proportion of HIV^+^ PWH who had ever experienced Hepatitis infection increased from 54% in 1988 to 95% in 2001 (Table 2). Because Hepatitis C was not identified until 1989, the measure from 1988 included Hepatitis B only. For HIV^+^ PWH, frequency of bleeding episodes tended to decrease from 1988 to 2012, but no significant trend was found compared to HIV^−^ PWH. In 2001 and 2012, almost all patients had severe hemophilia. The proportion of PWH in the sample having had inhibitor was below 25% in 1988 and 2001 but 33% in 2012. Generally, joint mobility declined over time for both HIV^−^ PWH and HIV^+^ PWH with only one patient from each group having full joint mobility (level 5) in 2012.

**Table 2:** Data characteristics by survey year and HIV status (%)

		1988		2001		2012	
		HIV^+^	HIV^−^	HIV^+^	HIV^−^	HIV^+^	HIV^−^
Age distribution	0–15 years	8	23	0	0	0	0
	16–24 years	26	21	6	6	6	6
	25–34 years	38	25	39	33	0	0
	35–44 years	11	21	39	39	44	33
	45–54 years	17	11	17	22	22	33
	55–88 years	0	0	0	0	28	28
	N	(53)	(53)	[18]	[18]	[18]	[18]
Factor use (per year)	0–25.000 units	8	12	0	0	6	6
	25.001–75.000 units	10	20	20	19	28	22
	75.001–125.000 units	22	16	35	38	11	22
	125.001–250.000 units	46	37	35	33	17	22
	250.001–500.000 units	8	12	10	5	33	22
	500.001- units	6	2	0	5	6	6
	N	(49)	(50)	[21]	[20]	[18]	[18]
Hepatitis B or C	Never	46^a^	63^a^	5	5	6	6
	Current or previous	54^a^	37^a^	95	95	94	94
	N	(46)	(46)	[21]	[21]	[18]	[18]
Bleeding episodes	No episodes	6	4	5	0	6	6
	1–10 episodes	34	53	53	50	61	33
	11–25 episodes	30	8	26	15	17	39
	26–50 episodes	19	21	16	25	11	11
	51+ episodes	11	15	0	10	6	11
	N	(53)	(53)	[20]	[19]	[18]	[18]
Severity of hemophilia	Severe	–	–	100	100	89	100
	Moderate			0	0	11	0
	N			[21]	[21]	[18]	[18]
Inhibitor (ever)	Never	77	84	81	90	67	67
	Current or previous	23	16	19	10	33	33
	N	(50)	(52)	[21]	[21]	[18]	[18]
Joint mobility	0	13	9	24	14	17	22
	1	17	17	10	5	6	22
	2	25	9	19	24	44	17
	3	15	21	10	24	17	22
	4	17	13	38	24	11	11
	5	12	30	0	10	6	6
	N						

^a^Only HBV

### Social function and well-being

**Fig. 1:**
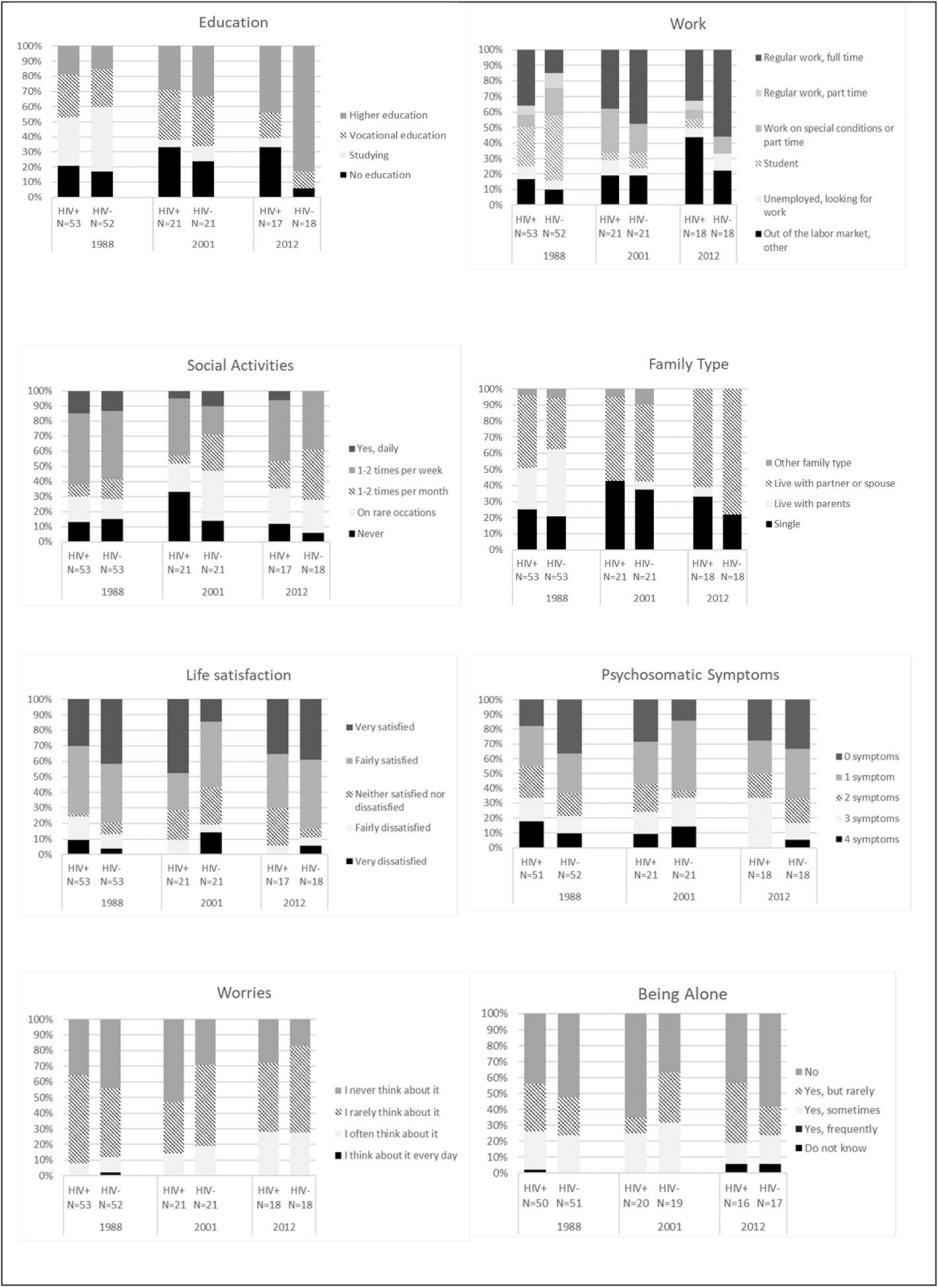
Descriptive information on variables

Table 3 shows tests of differences between HIV^+^ and HIV^−^ PWH for each of the 3 years and tests for trends over time for HIV^+^ PWH compared to HIV^−^ PWH. Figure 1 shows descriptive information for these variables. As shown in Table 3, three comparisons of HIV^+^ and HIV^−^ PWH were statistically significant at a 5% level; psychosomatic symptoms, life satisfaction and level of education. In 1988, HIV^+^ PWH had more psychosomatic symptoms than HIV^−^ (*p* = 0.026, Table 3, Fig. 1). In 2001, life satisfaction was higher among HIV^+^ compared to HIV^−^ PWH (*p* = 0,049, Table 3). In 2012, the level of education was higher among HIV^−^ compared to HIV^+^ PWH (*p* = 0,015, Table 3). Tests of differences in trend over time found two significant results: the trend in life satisfaction was more positive for HIV^+^ PWH compared to HIV^−^ PWH (*p* = 0,046, Table 3). Across years, HIV^−^ PWH attained higher levels of education than HIV^+^ PWH (*p* = 0,024, Table 3).

**Table 3:** P-values of trends statistically significant trends in *italic*

	HIV^+^/HIV^−a^			Trends (HIV^+^/HIV^−^)^b^
	1988	2001	2012	
Education	0,717	0,622	*0,015*	*0,024*
Work	0.075	0.534	0.328	0.113
Social activities	0.752	0.679	0.901	0.884
Family type	0,333	0,753	0,459	0,383
Life satisfaction	0.179	*0.049*	0.655	*0.046*
Psychosomatic symptoms	*0.026*	0.552	0.395	0.173
Worries	0.996	0.169	0.649	0.379
Being Alone	0.535	0.166	0.561	0.121

^a^Comparison between HIV^+^ and HIV^−^ for each year

^b^Trend over time for HIV^+^ compared to HIV

While no other statistically significant HIV-related differences were found, some trends in the data are worth noticing. While the proportion of HIV^+^ PWH with a regular full-time job was steady throughout the 24 years (range 33–38%), the proportion of HIV^−^ PWH with a regular full-time job increased from 15 to 56%. A decrease was seen in social activities, but this decrease was seen for both HIV^+^ PWH and HIV^−^ PWH. Similarly, an increase was seen in the number of both HIV^+^ PWH and HIV^−^ PWH living with partner or spouse, more prominently among HIV^−^ PWH.

### Stigmatization and openness about HIV

**Fig. 2:**
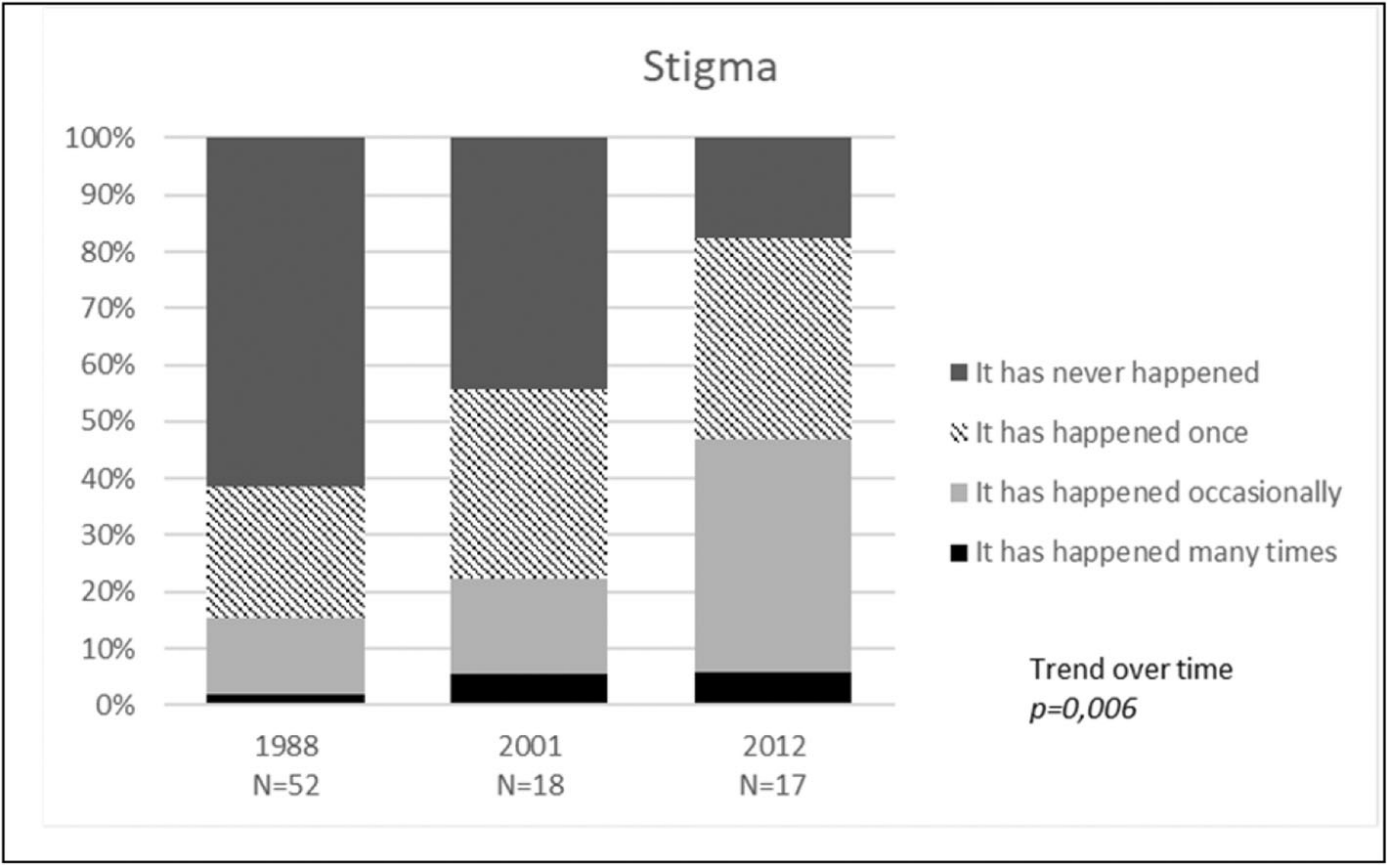
Perceived stigmatization of HIV status among HIV-positive PWH

**Fig. 3:**
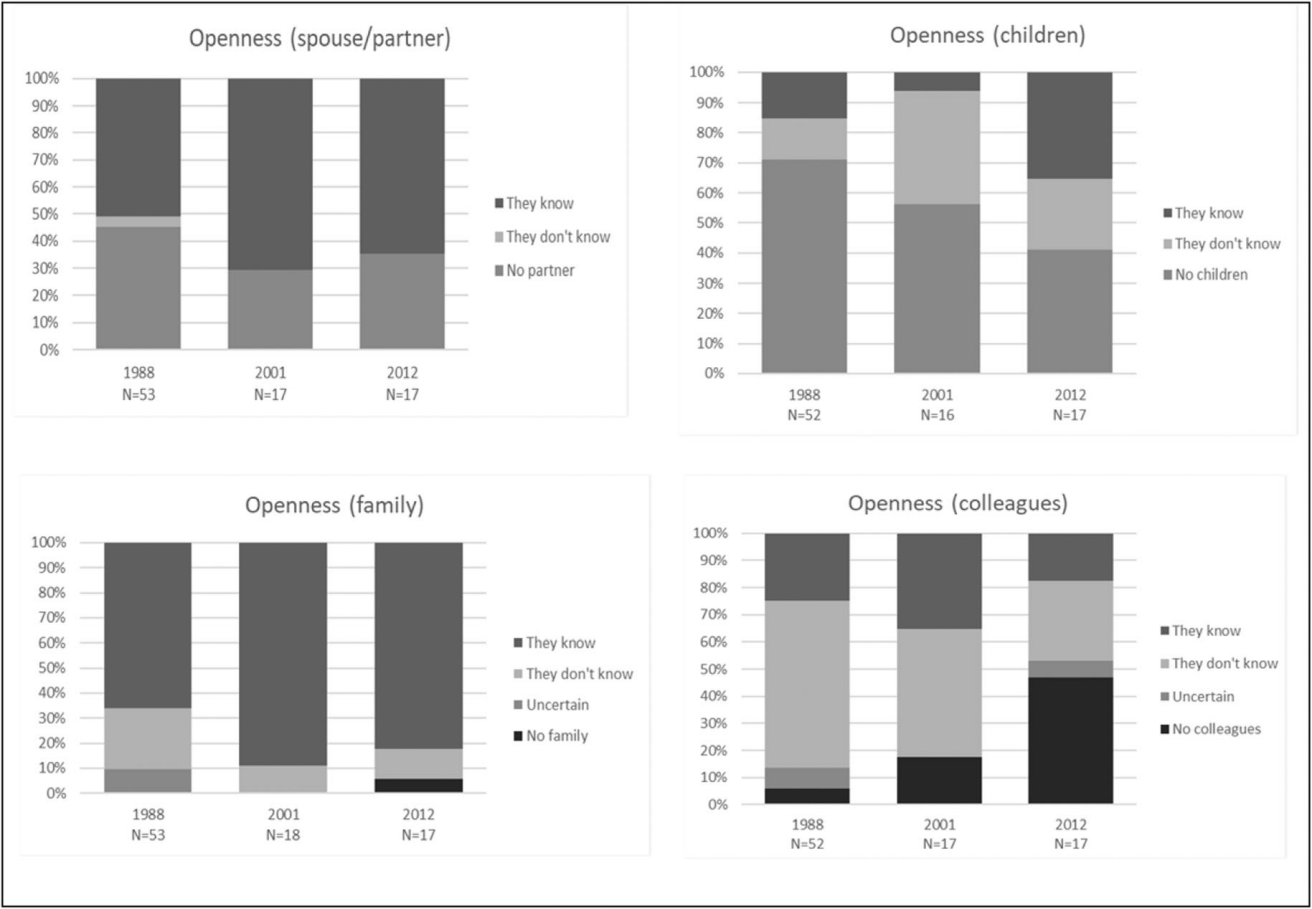
Openness about disease

Figures 2 and 3 show the analyses of the experienced stigmatization and openness towards spouse/partner, children, families and colleagues. There was a significant increase in experienced stigmatization among HIV^+^ PWH from 1988 to 2012 (*p* = 0,006, Fig. 2), with larger proportions of HIV^+^ PWH in 2012 that had experienced stigmatization *many times* or *occasionally*.

While no changes over time regarding openness were statistically significant, the figures suggest some trends: The proportion of HIV^+^ PWH being open about their HIV status to *spouse/partner* and *children* saw an overall increase from 1988 to 2012 (Fig. 3). In 2012 24% of HIV^+^ PWH indicated that their children did not know about their HIV status. Openness about HIV status to *other family* increased, whereas openness towards *colleagues* decreased.

## Discussion

This study found a higher burden of psychosomatic symptoms such as anxiety, headache, tiredness and depression among HIV^+^ PWH compared to HIV^−^ PWH in 1988. We also found higher levels of life satisfaction among HIV^+^ PWH in 2001 compared to HIV^−^, and a significantly more positive trend over time in life satisfaction among HIV^+^ compared to HIV^−^ PWH. In 2012, a higher level of education was seen among HIV^−^ PWH compared to HIV^+^ PWH and a significant trend over time towards higher education for HIV^−^ PWH. No differences between HIV^+^ and HIV^−^ PWH in social activities, feelings of loneliness, or worries about bleeding were found. Finally, in analyses restricted to HIV^+^ PWH, we found an increase in perceived stigmatization from 1988 to 2012.

Our findings on experienced stigma contradicted our hypotheses and were inconsistent with previous studies on stigma among HIV^+^ patients in general [9]. There are several possible explanations for these findings. A qualitative study from 2015 found HIV-related stigma within health contexts to be a broad and complex phenomenon [8]. Our questions may have been too simple to reflect this complexity. We asked about ever having experienced stigmatization, and the results from 2001 and 2012 may therefore reflect accumulation of experience through many years of being HIV infected. The fact that HIV^+^ PWH still recalls experience of stigmatization may reflect a persistent stigma associated with HIV infection and points to an issue of importance to HIV^+^ PWH.

We found weak and statistically insignificant trends towards greater openness about HIV status. A study from 2002 found that openness about HIV status among adolescents was positively associated with both social support, self-competence and decreased problem behavior [15]. The direction of an association between HIV openness and support is unclear. It is possible that openness about HIV induce sympathy and social support. On the other hand, HIV^+^ PWH may only choose to be open about HIV if they trust the environment to be supportive.

Our results regarding increased psychosomatic symptoms in 1988 are in line with a 1990 US study [11] and a 1992 Canadian study [5] finding elevated scores of depression and anxiety among HIV^+^ PWH. In relation to our findings on increased well-being among HIV^+^ PWH in 2001, results from other studies are mixed. Some studies show low satisfaction [5, 12, 13] and QoL [14]. An Italian study from 1995 [18] found worse psychological problems among 21 HIV^−^ PWH than among 24 HIV^+^ PWH and a study from 1999 found no association between HIV-status and health related QoL among individuals with severe hemophilia [16]. Qualitative data suggest that HIV^+^ PWH suffer from a psychological and physiological burden of HIV [22], as expressed by one Danish HIV^+^ PWH (authors translation) [23]:

*I still don’t dare to look far ahead into the future. For many years, my life revolved on getting used to dying soon, and now one suddenly has to live. In certain contexts, life is harder than death when you have grown up hand in hand with death.*

All results must also be interpreted in the light of scientific and social developments regarding hemophilia and HIV/AIDS over the 24-year span. In 1988, there was no curative treatment for HIV, thus HIV^+^ PWH were influenced by numerous uncertainties on their prognosis. Following the introduction of Highly Active Antiretroviral Treatment (HAART) in the late 1990s the prognosis for HIV^+^ PWH improved considerably which may explain our results showing increased life satisfaction among HIV^+^ PWH in 2001. The somewhat similar level of well-being between HIV^+^ PWH and HIV^−^ PWH in 2012 might be due to the two groups’ comparable disabilities and possibilities in life.

The differences between HIV^−^ and HIV^+^ PWH regarding educational level in 2012 may indicate that being co-infected with HIV has been a barrier for completing further education. The explanations for this barrier may be complex. HIV^+^ PWH, who were in the educational system in 1988 may have dropped out or decided not to pursue higher education in light of their perceived low life expectancy. In subsequent years where life expectancy dramatically improved for HIV^+^ PWH, they may have experienced ‘survivors guilt’ [24] and felt undeserved of pursuing opportunities such as higher education, despite their life expectancy being the same as for HIV^−^ PWH.

In general, our original hypotheses on differences regarding well-being and social function between HIV^+^ PWH and HIV^−^ PWH were only partly met. While qualitative studies from 1985 to 1991 describe severe psychological impact of HIV-infection among PWH [5, 11], several other authors have found surprisingly small differences between HIV^+^ PWH and HIV^−^ PWH [14, 16–18]. Similarities between HIV^+^ PWH and HIV^−^ PWH may have several explanations.

First, results may be biased by non-response from the HIV^+^ PWH with the largest impact of HIV infection, causing us to underestimate the impact of HIV.

Second, from 1988 to 2001 mortality was high among HIV^+^ PWH and may have been particularly high for the HIV^+^ PWH with worst health and lowest quality of life.

Third, even when responding, PWH may underreport symptoms and problems. Qualitative studies have found that some PWH downplay their symptoms and problems as a coping mechanism [25].

Fourth, lack of statistical power due to small sample size may cause us to overlook differences in well-being that are important to patients.

Fifth, Danish HIV^+^ PWH received an economic compensation for having been infected with HIV [26] and psychological and social counseling services for HIV^+^ PWH were established by the Danish Hemophilia Society. The compensations and opportunities for support may have reduced the psychological consequences of HIV infection.

Sixth, in light of the multiple burdens and health risks experienced by PWH, the incremental impact of HIV infection after the introduction of HAART might have been smaller than anticipated by outsiders. A limitation in understanding the differences between HIV^+^ PWH and HIV^−^ PWH is that we do not have person-level information about HIV-related comorbidities or antiretrovival treatment. On population level we know that HAART was offered to all HIV^+^ PWH in 2001 and 2012 and accepted by nearly all.

Finally, analyses of openness and perceived stigma were carried out in the HIV^+^ group only. The average age of HIV^+^ PWH increased from 1988 to 2012, which may have biased the results, if perceptions of stigma vary between the young and the old.

There are several strengths to be noted in the current study. First, a large proportion of the Danish HIV^+^ PWH from 1988 to 2012 were included, providing knowledge from the group of Danish patients. Second, the information was obtained over a long time span providing more extensive insight than previous studies. Third, propensity score matching enabled us to identify the best possible comparison groups of HIV^−^ PWH.

## Conclusion

The differences between Danish HIV^+^ and HIV^−^ PWH regarding both well-being and psychosomatic symptoms seem to have evened out from 1988 to 2012, even though there are still differences regarding employment and education. Despite great improvements concerning treatment, public attitude and knowledge about HIV, HIV^+^ PWH still experience challenges. The health risks and the psychosomatic burdens associated with both hemophilia and HIV should be held in mind when reviewing the need for social, psychological and financial support in this patient group.

## Supplementary information

**Additional file 1.** Description of study questionnaire.

## Data Availability

The datasets generated and/or analyzed during the current study are not publicly available due the protection of personal information among the group of anonymous participants. Tabled data are available from JBB on reasonable request.

## Abbreviations

HIV: Human Immunodeficiency Virus
PWH: Patients with hemophilia
QoL: Quality of life
