# Supplementary material for: “Development in well-being and social function among Danish hemophilia patients with HIV: a three-wave panel study spanning 24 years”
Source: BMC Public Health. 2019 Dec 19;19:1714. doi: 10.1186/s12889-019-8062-9 (PMC6923887; doi:10.1186/s12889-019-8062-9)
Supplement: Supplementary file 1 — Additional file 1. Description of study questionnaire. [file 12889_2019_8062_MOESM1_ESM.docx]

| **Description of study questionnaire** | | |
| --- | --- | --- |
| **Background variables** | **Definition/Question** | **Grouping/Response categories** |
| Age | Age at January 1^st^ of 1988, 2001  and 2012 | 15-24; 25-34; 35-44; 45-54; 55-88 |
| Number of bleeding episodes treated with factor | 1988: 5 response categories  2001-2012: # of episodes | 0; 1-10; 11-25; 26-50; >50 |
| Yearly factor use^1^ | Units per year | 0-25.000; 25.001-75.000; 75.001-125.000; 125.001-250.000; 250.001-500.000; >500.000 |
| Severity of hemophilia^1^ |  | Moderate; Severe |
| Inhibitor (ever) ^1^ |  | Never; Current or previous |
| Hepatitis B or C (ever) ^1^ |  | Never; Current or previous |
| Hepatitis treatment | “Have you been treated for heptatitis?” |  |
| HIV Infection^1^ |  | Yes; No |
| Prophylactic treatment | Self-report | Yes, constantly; Yes, sometimes; No |
| Joint status | Questions on locations of bleedings within the past year. | No bleeds; Only one bleeding ; Several bleedings |
| **Variables** | **Definition/Question** | **Grouping/Response categories** |
| Education | Highest education completed | Primary/secondary school (≤10 years); High school (11-12 years); Vocational education (e.g. apprentice­ship or trade education); Higher education (theoretical education of any length) |
| Work | Questions on current employment,  work hours and social benefits. | Regular full time work; Regular part time work (≤30 hours) ; Work on special conditions^2^; Unemployed but looking for work; Out of the labor market; Studying; Other |
| Family type |  | Living with spouse or partner; Living alone; Other family type (e.g. living with parents or house sharing) |
| Sick days in past year | Due to hemophilia | 0; 1-5; 6-10; 11-30; 31-365 |
| Bed days in past year | Due to hemophilia | 0; 1-5; 6-10; 11-30; 31-365 |
| Social activities | “Do you attend meeting, clubs, or other activities outside work or school, including sports, evening school or the like?” | Daily; 1-2 times a week; 1-2 a month; Rarely; Never |
| Availability of help | “Do you have friends or acquaintances who you can easily ask for practical help?” | Yes, it is easy for me to get help; Yes, it is possible… but not easy; No/Don’t know |
| Being alone | “Are you ever alone, but want to be together with other people?” | No; Yes, but rarely; Yes, sometimes; Yes, frequently; Don’t know |
| Limitations caused by hemophilia regarding family choices | “Have you chosen not to have children due to your bleeding disorder or complications thereof?” | No; Yes; Not relevant |
| Limitations caused by hemophilia | “How does your life compare to persons of your age without hemophilia?” | I can live a completely normal life; I can live a normal life with minor limitations; My disease causes rather large limitations; Due to my disease a normal life is virtually impossible |
| Overall health | “All in all, what do you think about your health” | Excelent; Very good; Good; Not that good; Bad |
| Life satisfaction | “All in all, how satisfied or dissatisfied are you with your life as it stands today?” | Very satisfied; Fairly satisfied; Neither satisfied nor dissatisfied; Fairly dissatisfied; Very dissatisfied |
| Being open about HCV status | “Who knows that you are HCV positive?” | Spouse or partner; Colleagues or Classmates; Children; Other family; Closest friends |
| Pain(symptoms) | ^[[1]](#footnote-1)^a. Shoulder or neck  b. Back or lower back  c. Arms, hands, legs, knees, hips or other joints. | Yes, very bothered; Yes, slightly bothered; No, not bothered. |
| Anxiety(symptoms) | ^1^a. Fast heartrate  b. Anxiety, nervousness, unrest.  c. Troubles sleeping. | Yes, very bothered; Yes, slightly bothered; No, not bothered. |
| Depression(symptoms) | ^1^Depressed, in low spirit, unhappy. | Yes, very bothered; Yes, slightly bothered; No, not bothered. |
| Tiredness(symptoms) | ^1^Tiredness. | Yes, very bothered; Yes, slightly bothered; No, not bothered. |
| Coping with bleedings | “Which one of the following statements is true about you?’’ | If I suspect a bleeding, I use factor immediately; When I have a bleeding, I always contact my doctor/ hemophilia center. |
| Educational limitations | “Has your illness affected on your choice of education?” | Yes; No; Do not Know. |
| Medicine use | “Do you use any medication besides factor treatment?” | Daily; 1-3 times pr. Week; 1-3 times pr month; No. |
| Worries | “Patients with heamophilia have a curtain risk of developing life-threatening bleeds. Do you ever think about that? ” | I never think about that; I randomly think about that; I often think about that; I think about it every day. |

1. “Have you, within the last 14 days, been bothered with following pain og discomforts..” [↑](#footnote-ref-1)
